# Mapping of quantitative trait loci and mining of candidate genes for seed viability in soybean [*Glycine max* (L.) Merr.]

**DOI:** 10.3389/fpls.2024.1372037

**Published:** 2025-02-14

**Authors:** Manisha Saini, Raju R. Yadav, Rahul Kumar, Subhash Chandra, N. Krishna Kumar Rathod, Meniari Taku, Manu Yadav, Sudipta Basu, Ambika Rajendran, S. K. Lal, Akshay Talukdar

**Affiliations:** ^1^ Division of Genetics, Indian Council of Agricultural Research (ICAR)-Indian Agricultural Research Institute, New Delhi, India; ^2^ Tripura Centre, Indian Council of Agricultural Research (ICAR)-Research Complex for NEH Region, Lembucherra, India; ^3^ Regional Station, Indian Council of Agricultural Research (ICAR)-Central Institute for Cotton Research, Sirsa, India; ^4^ Division of Seed Science and Technology, Indian Council of Agricultural Research (ICAR)- Indian Agricultural Research Institute, New Delhi, India

**Keywords:** soybean, seed viability, QTLs, candidate genes, marker-assisted breeding, germination%

## Abstract

Global oilseed crop soybean [*Glycine max* (L.) Merrill] contains 18%–20% oil, 40%–45% protein, and countless nutrients vital for human health. It is grown worldwide for food, feed, pharmaceutical, and industrial applications. However, inherent loss of seed viability during ambient storage poses serious bottleneck in the production and maintenance of quality seeds. Understanding inheritance and mapping of quantitative trait loci (QTLs) for seed viability would help in designing breeding program for developing varieties with higher viability of the seeds. In this study, attempt was made to map QTLs and identify candidate genes for seed viability in soybean. A high-viable genotype EC1023 (>90% germination after 1 year of storage) was hybridized with VLS61, a poor storing genotype (<70% germination after 1 year of storage), and the F_1_ seeds were advanced to the next generation. The F_2:3_ seeds were subjected to accelerated ageing (AA) by exposing it to 41°C at 100% RH for 72h followed by viability testing through germination test. After AA test, the germination of the parental genotypes EC1023 and VLS61 were 40% and 14%, respectively, and that of the F_2:3_ seeds ranged from 4.16% to 71.42% indicating wide variability in the viability of the seeds. Genetic polymorphism studied with 517 SSR markers indicated the polymorphism between the parental genotypes to be 20.35%; however, distribution of the polymorphism was not uniform across the chromosomes; Chr. 14 had 30.00% polymorphism as against 7.14% on Chrs.12. Through inclusive composite interval mapping approach, 8 QTL for seed viability, namely, qSv-6.1 and qSv-6.2, qSv-7.1, qSv-8.1, and qSv-8.2, qSv-10.1, qSv-13.1, and qSv-17.1 were mapped on Chrs. 6, 7, 8, 10, 13 and 17, respectively. The phenotypic variation explained (PVE) by the QTL were 1.97%–11.10%. Two QTL, namely, qSv-7.1 (PVE = 11.10%) and qSv13.1 (PVE = 11.08%) appeared to be major QTLs for seed viability and rest minor ones. All QTL except qSv8.2 appeared to be novel. The mapped QTLs were validated in 40 inter-specific RILs with varying level of seed viability. The SSR marker Satt538 linked to the QTL qSv8.2 could successfully (70%) separate the highly viable RILs from the poor-viable RILs. Similarly, SSR markers Sat_316 and Sat_173 were 80%–85% successful in separating the high and poor viable RILs. Based on Protein Analysis Through Evolutionary Relationships (PANTHER), gene annotation information, and literature search, more than 500 candidate genes for seed viability underlying the mapped QTL were identified. The mapped QTL and the identified candidate genes will pave the way for marker-assisted breeding of soybean to generate genotypes with improved seed viability.

## 1 Introduction

Across the globe, soybean [*Glycine max* (L.) Merrill] (2*n* = 40) is the leading oilseed crop contributing about 25% of the edible protein and 50% of the food oil, which constitutes nearly 57% of total oilseed production of the world (Phansak et al., 2016). Soybean, also crowned as “Golden bean” and “miracle bean,” contains 40%–45% protein, which possesses almost all the amino acids required by the human body for its general growth and development (Ali, 2009; Chandra et al., 2022). Soybean also contains 18%–22% oil, which is considered as nutritious and healthy vegetable oil owing to its richness in poly- and mono-unsaturated fatty acids. Furthermore, cultivation of soybean enriches the soil through symbiotic nitrogen fixation and improves the soil health (Kumar et al., 2023). Soybean is also used in the preparation of diversified food and food ingredients such as full-fat soy flour, soymilk, soy-cheese, curd, ice cream, sprouted and roasted snacks, soy fortified bakery, soy protein concentrate, dietary fiber, single-cell protein, citric acid, margarine, and so forth (Kumar, 2005). Similarly, de-oiled cake of soybean is a protein-rich animal feed with higher demand worldwide.

In India, soybean is grown in about 11.44 mha with a produce of 11.20 mt (SOPA, 2021-2022), which is 43% of the India’s total oilseed and around 25% of the edible oil production. Despite large-scale production, the Indian soybean productivity of about 882 kg/ha is too low as compared to the world soybean productivity, that is, 3417 kg/ha (SOPA, 2021-2022). In addition to other factors, maintenance of seed quality including seed germination, viability, and vigour during ambient storage condition are the major obstacles in soybean quality-seed production (Nkang and Umoh, 1997). Soybean seed reaches its greatest potential for germination and vigour at its physiological maturity (Crookston and Hill, 1978), which subsequently declines gradually till harvest followed by rapid decrease thereafter (Surki et al., 2012). The loss of viability is far more critical in tropical and sub-tropical regions of the world to which India belongs (Bhatia, 1996; Hang et al., 2015). Owing to this harsh reality, the minimum germination of soybean seeds for certification in India has been kept at as low as 70% (Bhatnagar and Tiwari, 1990; Dargahi et al., 2014). In order to develop soybean varieties that can tolerate stresses and maintain the viability of the seeds during ambient storage, it is necessary to understand the mechanism of seed deterioration.

Seed viability in soybean is a complex factor, which is affected by a number of physical, physiological, and genetic factors, namely, mechanical damage during harvest (Zihad, 2013), field weathering (Bhatia, 1996), imbibition kinetics and electrolyte leaching (Kuchlan et al., 2010; Sooganna et al., 2016), hard seed coat (Kumar et al., 2019), seed coat cracking (Moïse et al., 2005; Shelar et al., 2008; Chu et al., 2023), small seed size (Hosamani et al., 2013), black seed coats (Liu et al., 2017), tight attachment of the seed coat to the cotyledons (Kuchlan et al., 2010), and so forth.

Diverse and inconsistent reports are available about the genetic control of seed viability in soybean. It is reported to be controlled by one gene (Kebede et al., 2014; Sun et al., 2015; Adsul et al., 2018) or more than one gene (Watanabe et al., 2004; Singh et al., 2008; Zhang et al., 2008; Dargahi et al., 2014). Dao and Ram (1999) reported involvement of one (3:1) and two (15:1) genes in managing seed longevity in soybean, whereas Kueneman (1983) reported the influence of maternal trait on maintenance of seed viability. Like Clerkx et al. (2004), we too found the seed viability to be controlled by more than one gene (Saini et al., 2023).

Reports are available about mapping of some major and minor QTLs affecting viability in the soybean seeds, namely, five QTLs for viability (*VIS1* through *VIS5*) by Watanabe et al. (2004), two QTLs *Ha1* and *Ha2* reported by Zhang et al. (2008), and three QTLs by Dargahi et al. (2014). Singh et al. (2008) reported four SSR markers, namely, Satt434, Satt538, Satt281, and Satt598, to be significantly associated with seed longevity trait in soybean. Hosamani et al. (2013) identified three SSR markers Satt371, Satt453, and Satt618 for seed storability. Similarly, Sooganna et al. (2016) found that SSR marker Satt423 could distinctly differentiate good storing soybean genotypes from the poor ones. Similarly, two QTLs for seed viability were reported by Kumar et al. (2019). The diversity of findings stem from the use of diverse study materials and variable approaches for viability testing. People mostly tested the viability of the seeds after storing in ambient condition, which is a time taking approach. The accelerated ageing (AA) test, on the other hand, is a quick approach mimicking the natural process (Egli et al., 1978; TeKrony et al., 1980; Hosamani et al., 2013; Sooganna et al., 2016). Therefore, in this study, we attempted to map the genetic factors affecting viability of soybean seeds using AA and validated the results in an interspecific Recombinant Inbreed line (RIL) population.

## 2 Materials and methods

### 2.1 Plant materials

**Figure 1:**
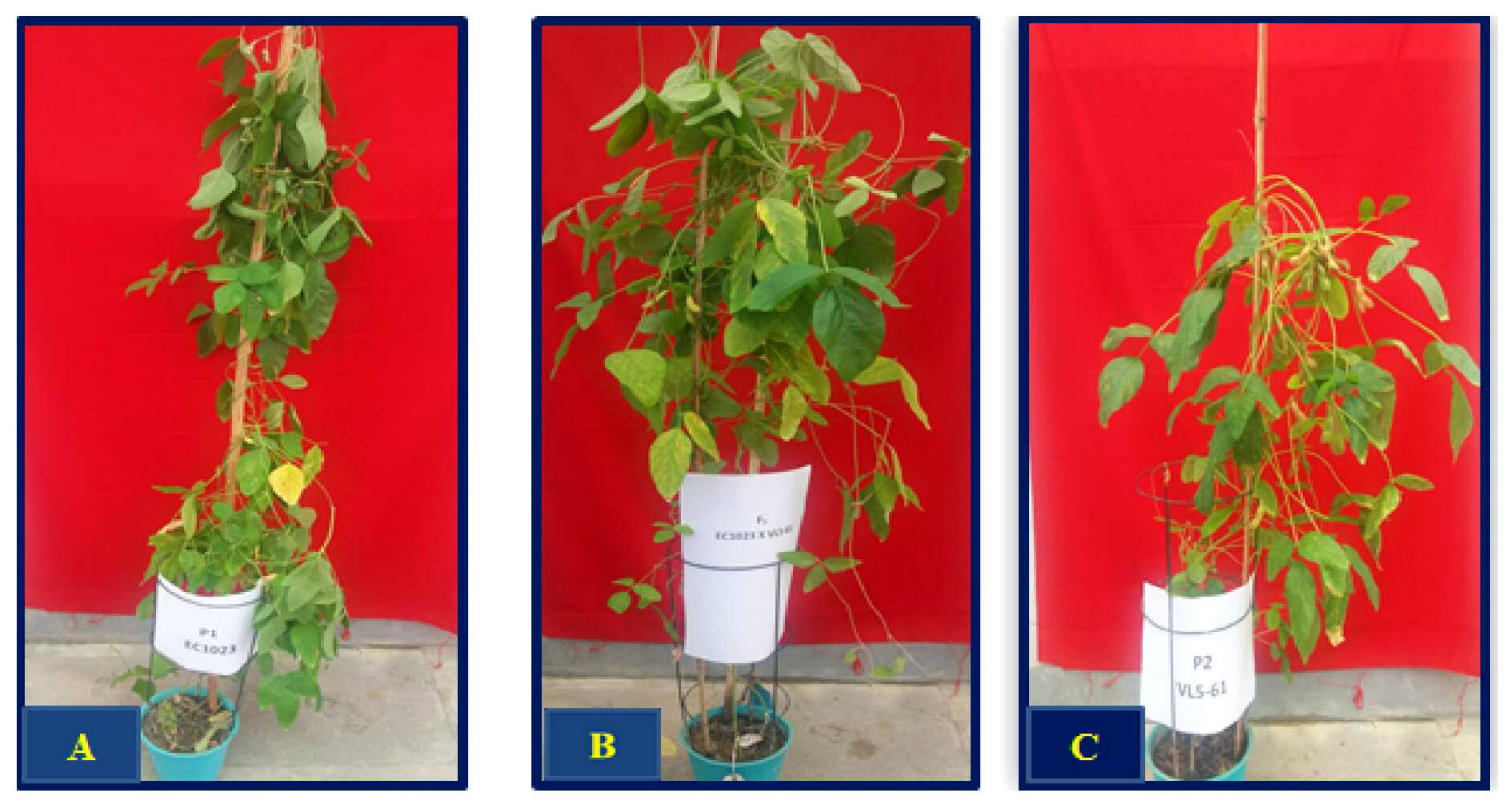
Morphological variation in the parental genotypes. **(A)** High-viable genotype: EC1023; **(B)** Hybrid (EC1023 xVLS61); **(C)** Poor viable genotype: VLS61.

The experimental material consisted of 125 intra-specific (*Glycine max*.) F_2:3_ population generated by hybridizing a highly viable genotype EC1023 (having 91.87% germination after 1 year of ambient storage) with VLS61 (a genotype with 60.87% germination after 1 year of ambient storage.) For effective hybridization, a novel technique, that is, pollination without emasculation as given by Talukdar and Shivakumar (2012) was utilized. The F_1_ and F_2_ seeds were grown under controlled conditions of the National Phytotron Facility, IARI, New Delhi and harvested separately and used in the mapping study. Contrasting features of both the parents (EC1023 and VLS61) along with their F_1_ are shown in **Figure 1**. Molecular analysis was done in 119 F_2:3_ plants, whereas phenotypic data could be collected from 49 plants as several plants died during the accelerated aging test. For validation of the findings of the present study, an interspecific RIL population was used, which was developed by crossing highly viable *Glycine soja* accession DC2008-1 with a high-yielding poor viable variety DS9712 (Yashpal et al., 2015). Out of 300 RILs, a set of 40 RILs (20 high viable and 20 poor viable) were used in this study.

### 2.2 Accelerated ageing test for seed viability

For testing seed viability, the seeds were subjected to AA by exposing it to 41°C for 72h under ~100% relative humidity (ISTA, 2009; Saini et al., 2023). The AA seeds were shade dried for about 2h and then placed in wet blotting papers and kept in a seed germinator cabinet at 25 ± 1°C and ~95% RH for 7 days (ISTA, 2009; Saini et al., 2023). On the 8th day, different parameters were recorded and germinated seeds were classified as normal seedlings, abnormal seedlings, hard seeds, and dead seeds. Degree of germination (%) was appraised as indicative of viability, that is, higher the germination (%), higher is the seed viability. However, in the present study we calculated seed viability in terms of the vigour of the seeds and seedlings. Thus, germination percentage was calculated from the normal seedling (having root to shoot ratio ~1) with good vigour which can give rise to a healthy plant. Germination data of the parental genotypes and the F_2:3_ populations were recorded separately and genotypes were classified as high viable (>40% germination), intermediate (30%–40% germination), and poor viable (<30% germination). Whereas, as per Indian Minimum Seed Certification Standards (IMSCS) (Tunwar and Singh, 1988), genotypes with ≥ 70% germination were classified as “high viable” and those with <70% germination were categorized as “poor viable”.

### 2.3 Phenotyping of the seed viability through ambient storage

For testing the viability of RILs lines, around 150 g seeds collected from the harvest of *Kharif* 2017 and 2018 were packed in water proof seed envelope and stored under ambient environment (average 25 ± 2°C and 65 ± 5% RH). Viability of the seeds was tested using between-paper method at 25°C in two replications of 50 seeds each following ISTA rules (Anonymous, 2013). The germination percentage was recorded on 8th day by counting the number of normal seedlings.

### 2.4 DNA extraction and molecular genotyping with SSR marker

Genomic DNA was isolated from tender soybean leaves using modified CTAB procedure (Lodhi et al., 1994). Quality and quantity of the DNA extracted from the mapping population was ascertained through spectrophotometer analysis. The DNA samples were diluted to a concentration of 20 ng/μl. Based on the consensus soybean genetic linkage map published by Cregan et al. (1999) and Song et al. (2004), SSR markers scattered throughout the 20 genetic linkage groups were chosen. A set of 506 SSR markers were used for the molecular genotyping of the parental genome, out of which 103 found to be polymorphic and were used for the molecular genotyping of the F_2_ population. Genomic DNA of 119 F_2_ plants were amplified by PCR and size separated in 3% metaphor gel through gel electrophoresis.

### 2.5 Linkage map construction and QTL mapping

For linkage map construction and to map the QTL for seed viability, software QTL IciMapping V4.2 was used. A genetic distance of 50 cM and a minimum LOD score of 2.5 was used to construct the linkage map connecting the markers. Kosambi’s mapping function (Kosambi, 1944) was used to calculate map distances. Method for QTL analysis was Inclusive Composite Interval Mapping of ADDitive (and dominant) QTL (ICIM-ADD) (Zeng, 1994). The phenotypic data, that is, germination (%) of the seeds of F_2:3_ progenies of the 49 F_2_ plants and the molecular genotypic data point of 97 SSR markers (of 103 polymorphic markers, six showed segregation distortion and hence discarded) were used to map QTL for seed viability. A LOD score of 2.5 was maintained to confirm the presence of a QTL in a particular genomic region. The threshold levels for each trait for ICIM-ADD mapping was computed by conducting a permutation test with 1000 permutations at 0.05 type -I error.

### 2.6 Allele mining and identification of candidate gene for seed viability

The QTLs identified and validated in this study were considered as a stable QTL. Model genes were downloaded from SoyBase (http://www.soybase.org) and EnsemblPlants (https://plants.ensembl.org) at the genomic location of the stable QTLs on the soybean genome (Glyma2.0). Gene ontology (GO) enrichment analysis was performed using Phytozome 13 (http://phytozome-next.jgi.doe.gov) for all the genes in each QTL region. The predicted candidate genes were then subjected to PANTHER Classification System in order to permit high-throughput analysis according to family and sub-family, molecular function, biological activity, and pathway.

## 3 Results

### 3.1 Phenotypic characterization of parents and F_2:3_ population for seed viability

Seed viability is the ability of the seed to produce normal healthy seedling after germination. After AA, the seed germination in EC1023 and VLS61 was 40% and 14%, respectively, which clearly showed the significant variations in viability between the two parental lines. The germination of the F_2:3_ seeds ranged from 4.16% to 71.42% with a mean of 17.31%. Similarly, seedling length, seedling dry weight, vigour indices I & II varied from 7.1–22.40 cm, 1.7–31.48 g, 6.6–1049.66, and 13.07–1694.88 with a mean of 17.45 cm, 10.76 g, 261.69, and 355.53, respectively. It was found that germination was positively and significantly associated with average seedling length (*r* = 0.78) and dry seedling weight (*r* = 0.83). Similarly, seedling length was found to be positively and significantly associated with dry seedling weight (*r* = 0.92). Based on germination percentage, the plants were classified as highly viable (>40% germination), intermediate (40%–30%) and poorly (low) viable (<30%). Out of the 49 plant progenies, nine were highly viable, two were intermediate, 21 were poorly viable and 17 progenies did not germinate at all (**Table 1**).

**Table 1:** Seed viability in the seeds of F_2:3_ progenies.

S. No.	Plant no.	Germination (%)	Viability status	S. No	Plant no.	Germination (%)	Viability status
1	C6 P-3	16.66	P	27	C10 P-6	0.00	P
2	C6 P-5	0.00	P	28	C10 P-7	4.16	P
3	C6 P-6	0.00	P	29	C10 P-8	27.5	P
4	C6 P-7	4.34	P	30	C10 P-9	15.38	P
5	C6 P-8	14.28	P	31	C10 P-10	5.26	P
6	C6 P-9	7.69	P	32	C10 P-11	41.66	H
7	C6 P-19	17.33	P	33	C10 P-12	18.18	P
8	C6 P-21	0.00	P	34	C10 P-13	14.28	P
9	C13 P-1	0.00	P	35	C10 P-14	0.00	P
10	C13 P-2	0.00	P	36	C10 P-15	40.00	H
11	C13 P-3	5.00	P	37	C10 P-16	0.00	P
12	C13 P-4	0.00	P	38	C10 P-20	33.33	I
13	C13 P-7	11.36	P	39	C10 P-21	15.00	P
14	C13 P-8	0.00	P	40	C10 P-22	17.64	P
15	C13 P-10	0.00	P	41	C10 P-24	37.83	I
16	C13 P-11	57.14	H	42	C10 P-25	25.00	P
17	C13 P-12	71.42	H	43	C10 P-26	47.82	H
18	C13 P-13	45.00	H	44	C10 P-27	0.00	P
19	C13 P-41	44.44	H	45	C10 P-28	20.00	P
20	C13 P-42	55.55	H	46	C10 P-35	53.84	H
21	C13 P-43	28.57	P	47	C10 P-36	0.00	P
22	C10 P-1	10.71	P	48	C10 P-37	0.00	P
23	C10 P-2	18.18	P	49	C10 P-42	23.33	P
24	C10 P-3	0.00	P	50	EC1023	40.00	H
25	C10 P-4	0.00	P	51	VL5-61	14.00	P
26	C10 P-5	0.00	P				

P, Poor viability (<30% germination); I, Intermediate (30%–40% germination); H, High viability (>40% germination).

The seed germination in the F_2:3_ seeds, which ranged from 14% to 40%, showed a continuous distribution indicating involvement of more than one gene in control of seed viability. The greater number of phenotypic classes and appearance of the transgressive segregants also indicative of involvement of more than one gene or QTLs and their recombination in the expression of the phenotypes.

### 3.2 Parental polymorphism survey

Genomic diversity of the parental genotypes at molecular level was evaluated with 517 SSR markers (@~25 markers per chromosome), which uniformly covered the entire soybean genome. Out of 517 SSR markers, only 103 were found to be polymorphic (19.92%). It was also observed that the chromosome-wise distribution of the polymorphic SSR loci was not uniform across the genome; some chromosomes had more polymorphic loci than others. Highest level of polymorphism (30.00%) was observed on chromosome number 14, while the least (07.14.78%) was observed on chromosome number 12 (**Table 2**).

**Table 2:** Chromosome-wise distribution of SSR markers used and their level of polymorphism in cross combination EC1023 xVLS61.

Chr. No.	Linkage Group	Total SSR used (No.)	Polymorphic SSR (No.)	Monomorphic SSR (No.)	Polymorphism Level (%)
1	D1a	25	6	19	24.00
2	D1b	34	4	30	11.76
3	N	22	3	19	13.63
4	C1	22	3	19	13.63
5	A1	33	8	25	24.24
6	C2	27	6	21	22.22
7	M	28	7	21	25.00
8	A2	26	3	23	11.53
9	K	24	6	18	25.00
10	O	27	5	22	18.51
11	B1	24	5	19	20.83
12	H	28	2	26	07.14
13	F2	33	9	24	27.27
14	B2	20	6	14	30.00
15	E2	17	4	13	23.52
16	J	28	5	23	17.85
17	D2	30	6	24	20.00
18	G	25	6	19	24.00
19	L	21	5	16	23.80
20	I	23	4	19	17.39
Total		517	103	414	19.92

### 3.3 Marker segregation analysis, map construction, and mapping QTLs for seed viability

As 103 polymorphic SSR markers were used for the molecular genotyping of the F_2_ population, the segregation data of each marker was subjected to chi-square (χ^2^) test for goodness of fit to 1:2:1 ratio. Out of the 103 polymorphic markers used, 97 markers showed goodness of fit to the expected 1:2:1 ratio, while six markers showed segregation distortion at a significance level of *P* < 0.05 and hence were excluded from further analysis. To map the QTL for seed viability, software QTL IciMapping V4.0 was used. Method for QTL analysis was Inclusive Composite Interval Mapping of ADDitive (and dominant) QTL (ICIM-ADD). The phenotypic data, that is, germination (%) of the seeds of F_2:3_ progenies of the 49 F_2_ plants and the molecular genotypic data point of 97 SSR markers were used to map QTL for seed viability. The threshold levels for each trait for ICIM-ADD mapping were computed by conducting a permutation test with 1,000 permutations at 0.05 type I error.

**Figure 2:**
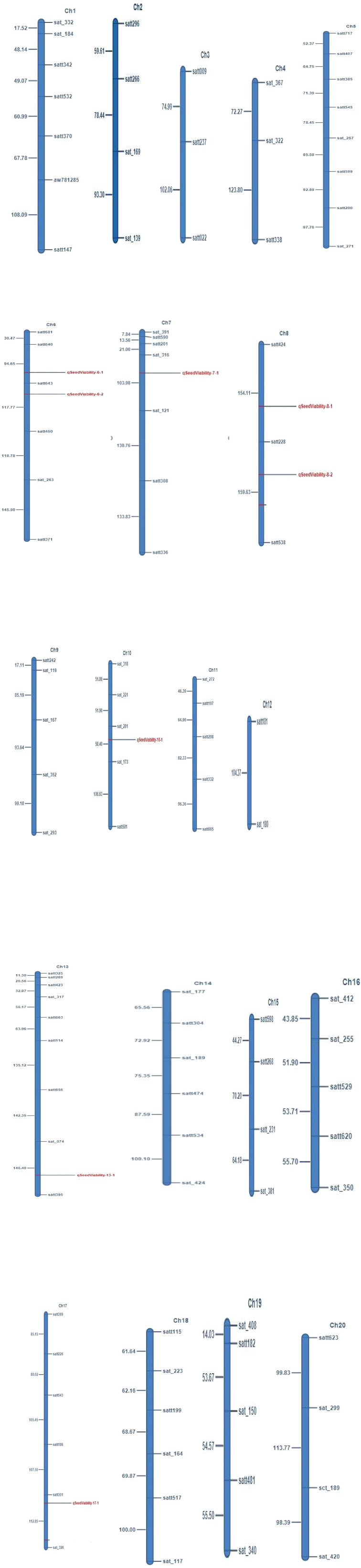
Linkage map of 20 soybean chromosome depicting the mapped QTLs for seed viability.

The marker-trait analyses mapped eight QTLs for seed viability on six different chromosomes. The QTL qSv-7.1 was mapped on chromosome number 7 and the phenotypic variation explained (PVE) by it was 11.10%. Similarly, qSv-13.1 was mapped on chromosome 13, which explained 11.08% of the phenotypic variations of seed viability. The QTL qSv-17.1 was mapped on chromosome 17 with 11.10% PVE. Two QTLs, namely, qSv-6.1 and qSv-6.2 were mapped on chromosome 6 that explained 2.72 and 2.68% of the phenotypic variations, respectively. Similarly, two QTLs, namely, qSv-8.1 and qSv-8.2 were mapped on chromosome 8 with respective 3.85% and 3.82% PVE values. One QTL qSv-10.1 was mapped on chromosome 10 that explained only 1.97% of the phenotypic variations of seed viability in the seeds. Thus, the range of PVE varied from 1.97 to 11.10% and the LOD of the QTLs ranged from 2.53 to 4.07. Map position of the identified QTL, markers bracketing them, phenotypic variance explained by the QTL, LOD and additive effect of the QTL are presented in **Table 3**. The linkage maps showing map position of the QTL have been depicted in **Figure 2**.

**Table 3:** List of QTLs detected for seed viability in soybean.

S. No.	Chromosome number	QTL	Map position (cM)	Marker interval (Lt marker-Rt marker)	LOD	PVE%	Additive effect	No of genes detected
1	7	qSv-7.1	77.02	Sat_316-Sat_121	4.07	11.10	0.94	65
2	13	qSv-13.1	557.23	Sat_074-Satt395	3.35	11.08	0.94	50
3	17	qSv-17.1	562.20	Satt301-Sat_326	3.06	9.53	−1.82	65
4	6	qSv-6.1	102.15	Satt640-Satt643	2.78	2.72	−1.10	82
5	6	qSv-6.2	155.15	Satt643-Satt460	2.93	2.68	−1.11	59
6	8	qSv-8.1	158.60	Satt424-Satt228	2.73	3.85	0.94	127
7	8	qSv-8.2	266.60	Satt228-Satt538	2.80	3.82	0.95	55
8	10	qSv-10.1	149.60	Sat_291-Sat_173	2.53	1.97	1.27	13

The QTL mapped on chromosomes 17 and 6 showed negative additive effects, which ranged from −1.82 to −1.10 (**Table 3**). It thus indicated that these alleles were contributed by the poor storing genotype, that is, VLS61. On the contrary, QTLs mapped on chromosomes 7, 13, 8, and 10 had positive effect ranging from 0.94 to 1.27, and might have come from the good-storing genotype, that is, EC1023 and had contributed towards enhanced viability of the seeds during ambient storage.

The QTL identified on chromosome number 8 coincides with the QTL reported earlier (Singh et al., 2008), and the rests appeared to be novel QTLs.

### 3.4 Validation study

#### 3.4.1 Phenotypic characterization of RIL population

Germination (%) in the fresh seeds of the *G soja* accession DC2008-1 and cultivated variety DS9712 was comparable, that is, 99% and 97%, respectively; however, it declined with the period of ambient storage more rapidly in the DS9712 than DC2008-1. Germination in the seeds of DC2008-1 was 96% and 92% after one and two years of ambient storage as compared to 70% and 51% in DS9712, respectively. Similarly, viability of the seeds of RILs varied significantly during storage and found to be 29%–97% and 1%–93% with a mean of 74.78% and 53.84% after one and 2 years of storage, respectively.

#### 3.4.2 Natural storage of seeds versus accelerated ageing test

RIL No. 7-33-2 had 74% germination after one year of storage, which got reduced to 4% after two years of ambient storage. Similarly, germination in the 1-year-stored seeds after AA was zero. Thus, the result of ambient storage and AA was comparable. Similarly, seeds of RIL No. 7-25-4 showed 50% and 48% germination after one year and two years of storage, respectively. The germination of the 1-year-old seeds after AA was 40% (**Table 4**). Both the results confirmed that the effects of ambient storage and AA are comparable.

**Table 4:** RILs with germination percentage with different storage periods.

S.no.	RIL no.	Hundred seed weight	Germination (%) after one year of storage	Germination (%) after 2 years of storage	Germination (%) after AA test
1	34-30-1	3.3	68	4	24
2	15-18-3	2.54	90	82	32
3	15-69-2	3.12	80	54	16
4	27-13-3	1.88	78	4	20
5	27-13-1	1.96	78	18	48
6	29-8-01	1.84	66	60	32
7	14-3-05	2.04	80	10	8
8	7-19A-2	1.48	84	56	28
9	6-4-3	2.14	76	12	28
10	7-22-30	1.92	72	44	24
11	7-33-2	2.94	74	4	0
12	8-17-4	1.54	72	36	28
13	8-21-5	1.52	70	18	16
14	15-6-1	2.30	68	62	8
15	32-1-4	2.06	88	52	4
16	2-37-1	2.26	78	46	8
17	34-21-4	2.02	68	34	16
18	2-37-4	1.74	82	52	16
19	2-34-1	3.08	86	46	8
20	2-34-3	2.32	80	54	32
21	34-4-5	1.82	36	34	24
22	14-15-1	1.90	52	46	32
23	34-4-2	1.78	36	20	24
24	34-32-2	2.70	34	18	24
25	15-50-1	2.82	52	18	4
26	16-72-5	1.68	28	20	0
27	17-1-02	1.80	26	10	24
28	34-9-3	1.82	40	6	16
29	13-40-4	2.14	20	16	16
30	31-1-03	3.04	56	30	8
31	13-37-2	1.86	16	18	12
32	13-63-5	2.20	24	18	4
33	13-49-4	2.22	28	26	16
34	7-18-2	2.96	40	20	24
35	17-1-05	1.60	16	26	12
36	7-26-1	2.36	30	30	12
37	34-30-7	2.22	62	6	0
38	15-33-5	2.50	58	46	32
39	7-25-4	2.02	50	48	40
40	8-21-3	1.8	32	24	8

#### 3.4.3 Validation of previously reported marker

**Figure 3:**
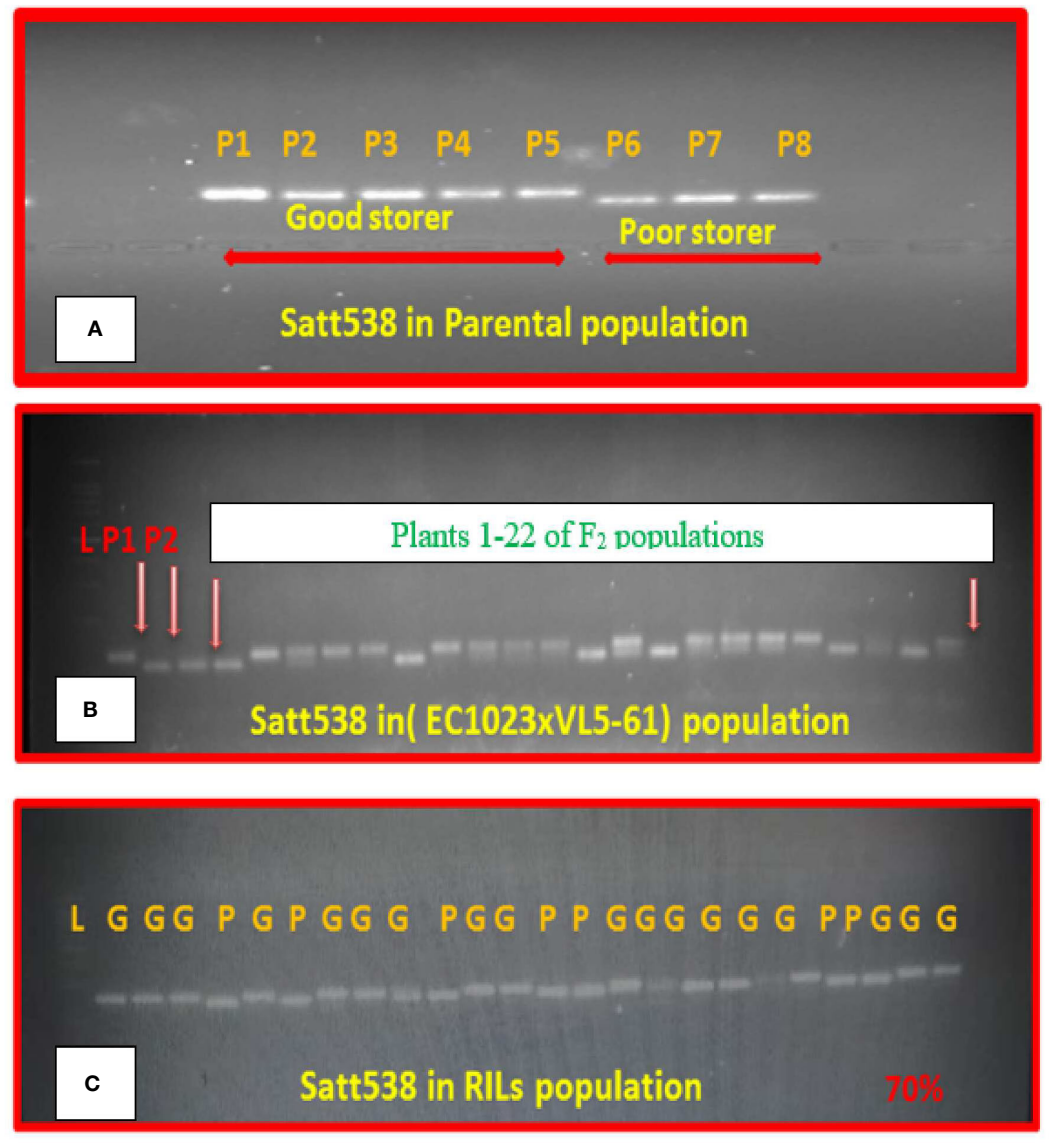
Segregation pattern of SSR marker Satt538 in **(A)** Eight parental genotypes. **(B)** F_2_ population. **(C)** 40 RIL lines.

While validating the linked markers reported earlier, only one SSR marker, that is, Satt538 located on chromosome 8 could effectively differentiate the five highly viable genotypes from the three poorly viable parental genotypes (**Figure 3A**). Banding pattern of Satt538 in the F_2_ plants of EC1023xVLS61 was depicted in a gel (**Figure 3B**). Similarly, the marker Satt538 could differentiate the good and poor storing RILs to about 70% correctly (**Figure 3C**).

#### 3.4.4 Validation of the novel reported markers in RILs population

**Figure 4:**
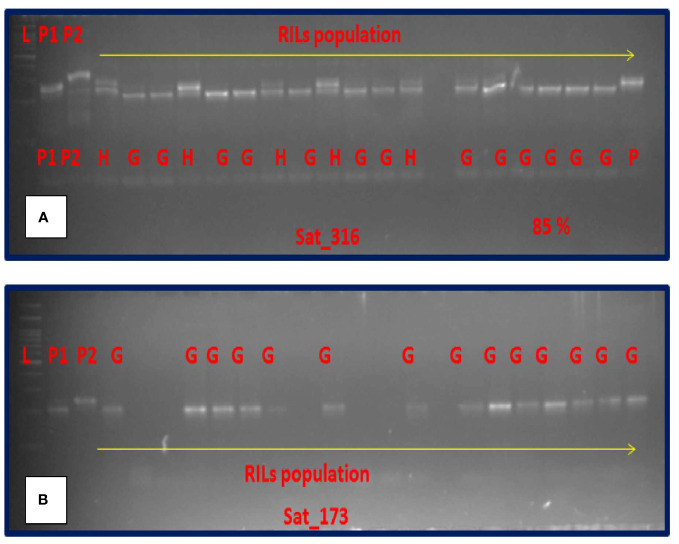
Amplification pattern of SSR makers in RIL population. **(A)** Sat_316. **(B)** Sat_173. [L = ladder, P1 = EC1023, P2 = VLS61, H = Heterozygous, G = Good storer.

Out of the 16 SSR markers flanking the 8 QTL mapped here, only two markers, namely, Sat_316 and Sat_173 could effectively separate the good and poorly storing RILs with a success rate of nearly 85% (**Figure 4**) and 80%, respectively.

#### 3.4.5 Gene ontology and candidate gene prediction within stable QTL

In the present study, eight QTLs, namely, qSv-7.1, qSv-13.1, qSv-17.1, qSv-6.1, qSv-6.2, qSv-8.1, qSv-8.2, and qSv-10.1 were mapped on six different chromosomes, and the QTL, qSv-8.2 with flanking markers satt228-satt538 was validated in the RIL population. The GO and candidate gene prediction analysis was performed for the mapped QTLs. Within the physical genomic interval of qSv-7.1, qSv-13.1, qSv-17.1, qSv-6.1, qSv-6.2, qSv-8.1, qSv-8.2 and qSv-10, a total of 65, 50, 65,82, 59, 127, 55, and 13 model genes were found to be present, respectively. The candidate genes were downloaded from SoyBase ((http://www.soybase.org) and EnsemblPlants (https://plants.ensembl.org). For the gene annotation which were found in each QTL Phytozome 13 was used (**Table 3**). The eight QTLs each had an increased number of genes associated with cell organelles, catalytic activity, binding, metabolic processes, and cellular processes, suggesting the critical role of these functions in the growth of soybean seeds (**Supplementary Material**).

## 4 Discussion

In tropical and sub-tropical regions, including India, sustention of soybean seed viability until subsequent planting is one of the principal constraints in the soybean cultivation (Hang et al., 2015). Declination in seed viability begins after physiological maturity (Crookston and Hill, 1978) followed by fast declining during ambient storage (Surki et al., 2012), which may even go down to zero in 10 months of storage (Bhattacharya and Raha, 2002). Poor longevity of the soybean seeds not only affects seedling vigour, crop stand in the field, and ultimately the seed yield (Zhang et al., 2019) but also increases the extra seed requirements and corresponding cost of cultivation. Therefore, improving seed viability in soybean is important factor to increase overall crop production (Dargahi et al., 2014). Studies attempting to figure out the component(s) responsible for viability loss hinted that seed viability is quite complicated. It is influenced by seed characteristics, namely, seed size, colour, permeability (Kumar et al., 2019), seed composition, integrity of the seed coat, mechanical damage, field weathering, and environmental factors such as moisture content, relative humidity, oxygen pressure, temperature of storage (Potts et al., 1978; Groot et al., 2012) in addition to the oil and moisture contents. The factors causing loss of viability becomes more damaging with the increased period of ambient storage; however, it varies with genotype, species and other varietal characters (Kurdikeri et al., 2000). Thus, enhancement of the seed viability in soybean through molecular breeding approach is the one of the most economically efficient long-lasting solution. In the present study, attempts were made to identify the molecular markers, QTLs and candidate genes linked to the seed viability in the soybean.

### 4.1 Inheritance of seed viability

In the present study, seeds of a F_2:3_ population derived from a cross between EC1023 and VLS61 were subjected to AA followed by germination test (ISTA, 2009; Dargahi et al., 2014; Saini et al., 2023). The germination in the population ranged from 4.16% to 71.42% indicating existence of wider variability in the seeds for viability. The range of seed germination in the F_2:3_ seeds (4.16%–71.42%) went beyond the range of germination of parental genotypes, that is, 14% and 40%, which indicated transgressive segregation. It has been shown earlier that while plotting the germination data in frequency distribution diagram, it showed continuous distribution keeping the parental data within the range (Saini et al., 2023). It thus indicated involvement of polygenes called quantitative trait loci (QTL) in controlling the seed viability trait in soybean (Saini et al., 2023). It was supported by the wider variations in germination of the seeds and appearance of the transgressive segregants for viability traits. Verma and Ram (1987) reported involvement of two to four genes for seed longevity in soybean. Using RFLP markers, Keim et al. (1990) identified five genomic regions contributing towards hard-seededness, which also affects seed germination and viability. Clerkx et al. (2004) indicated the seed viability to be a complex trait controlled by several genes and affected by environmental conditions during seed formation, harvest and storage. Hosamani et al. (2013) reported a set of linked SSR markers and indicated that genetic makeup of soybean genotypes has a role in determining the viability of the seeds during storage.

### 4.2 Mapping of QTLs for seed viability

Identification of molecular markers tightly linked to the gene/QTL governing seed viability and their deployment could offer a long-lasting solution to the problem of rapid viability loss in soybean (Zhou et al., 2010; Kumar et al., 2019). For mapping of gene/QTL, SSR or microsatellite markers are preferred over others because of its co-dominant nature of inheritance, high reproducibility, random distribution in genome, abundance and multi allelic nature (Saghai Maroof et al., 1994; Gupta and Varshney, 2000). In the present study, SSR markers covering the entire genome of soybean were selected for construction of linkage map and mapping of QTL for seed viability. For QTL mapping, diverse types of mapping populations, namely, F_2_, BC, DH, RIL, NIL, etc. can be utilized (Paterson, 1996); however, each population has its own strength and weaknesses (Singh and Singh, 2015). Similarly, F_2:3_ populations are also suitable for mapping of genes/QTL as it allows recording of data on multiple plants in each F_2:3_ family to compensate the sampling error. The mean phenotypic values from multiple plants in a F_2:3_ families are considered to be representative of the phenotype of its parental F_2_ plant (Yu et al., 1997). In the present study, a set of F_2:3_ progenies derived from a cross between EC1023 and VLS61 were used to map QTL for seed viability. F_2:3_ populations were also successfully used by Singh et al. (2008); Dargahi et al. (2014) and Adsul et al. (2018), to captures higher phenotypic variations of the target trait enabling detection of large effect QTL for effective deployment in marker assisted selection.

Genetic polymorphism between the parental genotypes (EC1023 and VLS61) studied through 517 SSR markers indicated to be 19.92%. Both the parental genotypes being from the same species *Glycine max*, lower level of polymorphism was inevitable. Singh et al. (2008) found 21 out of 145 SSR markers to be polymorphic with 14.48% polymorphism between a pair of cultivated soybean genotypes. However, Dargahi et al. (2014) observed 46% polymorphism in a set of soybean genotypes. Similarly, Kumar et al. (2011) reported 43.38%–48.38% polymorphism in soybean. Naik et al. (2019) recorded 53.33% polymorphism in soybean. Thus, the level of polymorphism found to vary with the type of genotypes used for the study. Polymorphism is generally high in inter-specific genotypes. Liu et al. (2017) observed about 64.38% polymorphism between Tokai-780 (*G. max*) and Hidaka-4 (*G. soja*) genotypes. Similarly, Kumar et al. (2019) reported 52.9% level of polymorphism between DC2008-1 (*G. soja*) and DS9712 (*G. max*), in which 164 out of 310 SSR markers used were polymorphic between the parents. The level of polymorphism thus represents genetic distance between the tested genotypes (Apuya et al., 1988). However, the distribution of polymorphism may not be uniform across the genome. In the present study, the highest level of polymorphism (30.00%) was observed on chromosome number 14 and the least (07.14. %) was on chromosome numbers 12. Kumar et al. (2019) also reported the non-uniform distribution of markers across the chromosomes. Soybean is a paleopolyploid. Early genome duplication followed by recombination- even or uneven- might have created variation across the genome.

In a segregating population, any deviation of observed frequencies from their expected mendelian frequencies of an individual in a given genotypic class has been defined as segregation distortion (Sandler and Golic, 1985; Lyttle, 1991; Kumar et al., 2019). It usually occurs in almost all the mapping populations with diverse intensities; however, intraspecific F_2_ population are expected to show relatively lower frequencies of distorted markers (Yamagishi et al., 2010). In the intra-specific F_2_ population used in this study, 6 out of 103 polymorphic markers exhibited distorted segregation (5.82%), while 16.4% was reported in an inter-specific RILs by Kumar et al. (2019). Segregation distortion may occur due to scoring error, gametic or zygotic selection, chromosome rearrangement, genetic incompatibility, pollen competition, preferential fertilization, etc. However, differential gametophytic selection is considered to be the primary cause of segregation distortion in rice (Xu et al., 1997).

For identification of gene/QTL controlling a trait of importance, high-density linkage map plays a major role (Tanksley et al., 1989; Mohan et al., 1997). The linkage map constructed in the present study contained 97 SSR markers distributed across the 20 chromosomes of soybean genome. Total length of the genetic map constructed in this study was 2287.87 cM with an average marker distance of 16.6 cM. Liu et al. (2007) constructed a linkage map with 282 markers that covered 2383cM and had 8.5cM average distance between the markers. Similarly, Li et al. (2008) constructed a genetic map of 1073.9cM long with an average marker density of 7.9cM. Zhang et al. (2008) used 148 markers to construct a linkage map of 1363.7cM length while studying the seed viability. For mapping water uptake trait in soybean, Molnar et al. (2012) constructed a linkage map of 2645 cM covering 20 chromosomes with 277 SSR markers. The variations in the map length are the result of a number of factors including number of markers used for the linkage map construction, segregation pattern of the markers, missing values, accuracy of the linkage analysis, marker density, etc (Castiglioni et al., 1999). For precision mapping, it is important to use large population and high-density linkage map (Kumawat et al., 2012). The linkage map generated in this study had good number of markers and nearly uniform distribution of markers across the chromosomes and hence could effectively map QTLs for seed viability and seed weight.

For QTL mapping, software ICIM 4.1.00 and composite interval mapping (CIM) approach was used. Commonly, a fixed LOD is utilized in QTL mapping; however, in the current study a threshold level was calculated separately for each case through permutation-combination test with LOD value greater than 2.5. For mapping QTL, genotyping was done in F_2_ population and phenotyping was performed in F_2:3_ progenies. For seed viability, 8 QTL were mapped on 6 chromosomes, namely, Chr. 6, 7, 8, 10, 13 and 17. On Chromosome 7, 10, 13, and 17, only one QTL each was mapped, while two QTL each was mapped on Chr. 6 and 8. The PVE by the QTL ranged from 1.97-11.10% and the LOD of the QTLs mapped ranged from 2.53 to 4.07. The QTL qSv-7.1 and qSv13.1 having 11.10% and 11.08% PVE were considered as major QTLs. Singh et al. (2008) mapped QTL for seed viability on the same region on Chr.8 where a QTL has been mapped in this study. Consistency in appearance, higher PVE and confirmation with past reports validated the QTL on Chr.8. Other QTLs for viability appeared to be novel as no QTL has yet been mapped in these regions. PVE and consistencies in expression are the two important factors for applicability of QTL in plant breeding. A stably expressing QTL is to be preferred over an unstable QTL even if its effect is moderate (Liu et al., 2017). The consistent QTL mapped in this study may be used in breeding program for enhancing viability of seeds in soybean. Previously, five QTLs for viability (*VIS*1-5) by Watanabe et al. (2004), two QTLs *Ha1* and *Ha2* by Zhang et al. (2008), three QTLs by Dargahi et al. (2014) and two QTLs for seed viability were mapped by Kumar et al. (2019). Association of SSR markers, namely, Satt434, Satt538, Satt281 and Satt598 (Singh et al., 2008), and Satt371, Satt453 and Satt618 (Hosamani et al., 2013) with seed storability have been reported. Sooganna et al. (2016) reported that SSR marker Satt423 could distinctly differentiate good-storing soybean genotypes from poor ones. In contritely, Adsul et al. (2018) reported single major gene with some other genes for seed longevity. Permeable seeds are relatively less viable than impermeable ones. Sun et al. (2015) identified a base substitution (T→G) in a gene (*GmHs1-1*) associated with calcium content in the seed coat that transformed the impermeable seed coat to permeable ones. Jang et al. (2015) made a similar observation.

### 4.3 Accelerated ageing and validation of linked markers in interspecific RIL population

Validation of identified QTLs in a set of unrelated germplasm or mapping population is highly desirable in determining its efficacy and usefulness in breeding program. For validation of the previously reported QTLs for seed viability as well as QTLs identified in the present study, an inter-specific RIL population developed by crossing poor storing genotype DS 9712 [*Glycine max* (L.) Merr.] and good-storing genotype DC 2008-1 (*Glycine soja* Sieb. & Zucc.) was used. This population was enormously diverse for several traits (Yashpal et al., 2015) including yield and components and included transgressive segregants for most of the traits (Rathod et al., 2019). For validation study, 40 lines were selected on the basis of their germination, 20 were good storer and 20 were poor. The RILs varied for seed viability after periods of ambient storage and AA treatment. Testing viability of seeds through ambient storage is a time taking process. Contrarily, AA mimicking the ambient storage is a rapid and effective approach of viability testing in seeds including soybean. Artificial exposure of the seeds to higher temperature and humidity for prescribed time period provide the simulation results with natural ageing (Egli et al., 1978; TeKrony et al., 1980). The RIL No. 7-33-2 showed 74% germination after one year of storage whereas it declined to 4% germination after two years of ambient storage. Germination in the one-year stored seeds after AA was zero. Similar results were also found in RIL No. 15-50-1; however, exception could not be ruled out. Thus, effect of AA on seed viability was comparable to that of one-year ambient storage. Similar observation was reported by Egli et al. (1978), TeKrony et al. (1980), and Dargahi et al. (2014). Effectiveness of AA in testing viability was proved in several other crops including mungbean (Bishnoi and Santos, 1996) and chickpea (Dahiya et al., 1997).

The 8 QTL mapped in this study for seed viability were flanked by 16 SSR markers. The marker Satt538 could effectively separate high-and low-viable genotypes. Singh et al. (2008) also found similar result. Segregation analysis of marker Satt538 on the selected 40 RILs was 70% successful in separating the good storer genotypes from the poor storer ones. Thus, this marker would be useful in identification of soybean genotypes with high viability. Similarly, two other markers, namely, Sat_316 and Sat_173 was 80%–85% successful in separating the good storing RILs from others. Other SSR markers reported to be linked to seed viability including Satt538, Satt285, Satt600, and Satt434 (Singh et al., 2008), Satt371, Satt453, and Satt618 (Hosamani et al., 2013), Satt423 (Sooganna et al., 2016), and Satt281 (Naik et al., 2019) were also tested. Additionally, the QTL qSv8.2 was validated in the RILs. Of late, genotype-by-sequencing and genome-wide association studies approaches are being utilized to validate genomic loci associated with qualitative and quantitative traits in soybean (Sonah et al., 2015). In this study, none of the minor QTLs could be validated in the inter-specific RILs. Such results are also not uncommon, as some QTL might be specific for a specific mapping population (Vasilia et al., 2004; Radhika et al., 2007). It may also happen because of the background effect or because of harbouring alleles different from the one in original mapping population (Palomeque et al., 2010). The validation study confirmed involvement of major QTL in controlling the seed viability in soybean. The markers validated in the independent population should be useful for improving seed viability in soybean through molecular breeding.

### 4.4 Mining of the candidate genes for seed viability

To improve the desired trait through breeding, it will be necessary to identify the actual candidate gene that falls beneath the QTL region. The present work identified potential candidate genes for soybean seed viability by employing data from the literature that was available, gene annotation, and bioinformatics methods. With the similar analysis Kumar et al. (2023) reported 66 genes for seed size and shape in soybean. From seven stable QTLs, Karikari et al. (2019) extracted 66 of the 381 potential genes are mostly associated with cellular components, catalytic activity, transportation, metabolic, and cellular processes, all of these being essential for seed development. The identified candidate genes have either a direct or an indirect function in regulating the soybean seed viability, seed size, and shape as well as their growth and development via various mechanisms as cell component storage, lipid and protein storage, transport, metabolic processes, plant hormone signal transduction, ubiquitin-proteasome pathway degradation, and fatty acid beta-oxidation. Thus, by the use of these findings, strategies for increasing soybean production can be established comprehending functional networks. The candidate’s genes and markers identified in this study provide significant genetic resources for soybean. Finally, the primary and stable QTLs found in this study should be fine mapped in order to identify tightly linked markers for efficient molecular breeding aimed at enhancing soybean seed viability and yield.

## 5 Conclusion

Using seeds from a F_2:3_ segregating population derived from a cross between a high-viable and a poor storing soybean genotype, and their phenotypic characterization through AA test followed by germination test, it was found that the viability of seed is a complex trait and it controlled by more than one gene. Using SSR markers, eight QTLs were mapped on six chromosomes, of which two were major QTL. One previously identified marker and two currently identified markers could be validated in an inter-specific RIL population confirming their suitability in identification of soybean genotypes with higher seed viability. The AA test results were found to be comparable to that of ambient storage. The findings of this study will help the soybean breeders in breeding soybean for higher viability of seeds.

## Data availability statement

The original contributions presented in the study are included in the article/**Supplementary Material**, further inquiries can be directed to the corresponding authors.

## Author contributions

MS: Conceptualization, Data curation, Formal analysis, Investigation, Methodology, Software, Supervision, Validation, Writing – original draft, Writing – review & editing. RY: Conceptualization, Data curation, Methodology, Writing – review & editing, Writing – original draft. RK: Methodology, Validation, Writing – review & editing, Writing – original draft. SC: Data curation, Investigation, Supervision, Validation, Writing – review & editing, Writing – original draft. NR: Investigation, Methodology, Writing – review & editing, Writing – original draft. MT: Supervision, Validation, Writing – review & editing, Writing – original draft. MY: Investigation, Methodology, Writing – review & editing, Writing – original draft. SB: Formal analysis, Investigation, Methodology, Supervision, Writing – review & editing, Writing – original draft. RR: Data curation, Methodology, Supervision, Writing – review & editing, Writing – original draft. SL: Formal analysis, Project administration, Supervision, Validation, Writing – review & editing, Writing – original draft. AT: Conceptualization, Formal analysis, Project administration, Supervision, Writing – review & editing, Writing – original draft.
